# Supplementary material for: Chidamide increases the sensitivity of Non-small Cell Lung Cancer to Crizotinib by decreasing c-MET mRNA methylation
Source: Int J Biol Sci. 2020 Jul 19;16(14):2595–611. doi: 10.7150/ijbs.45886 (PMC7415423; doi:10.7150/ijbs.45886)
Supplement: Supplementary file 1 — Supplementary figures and tables. [file ijbsv16p2595s1.pdf]

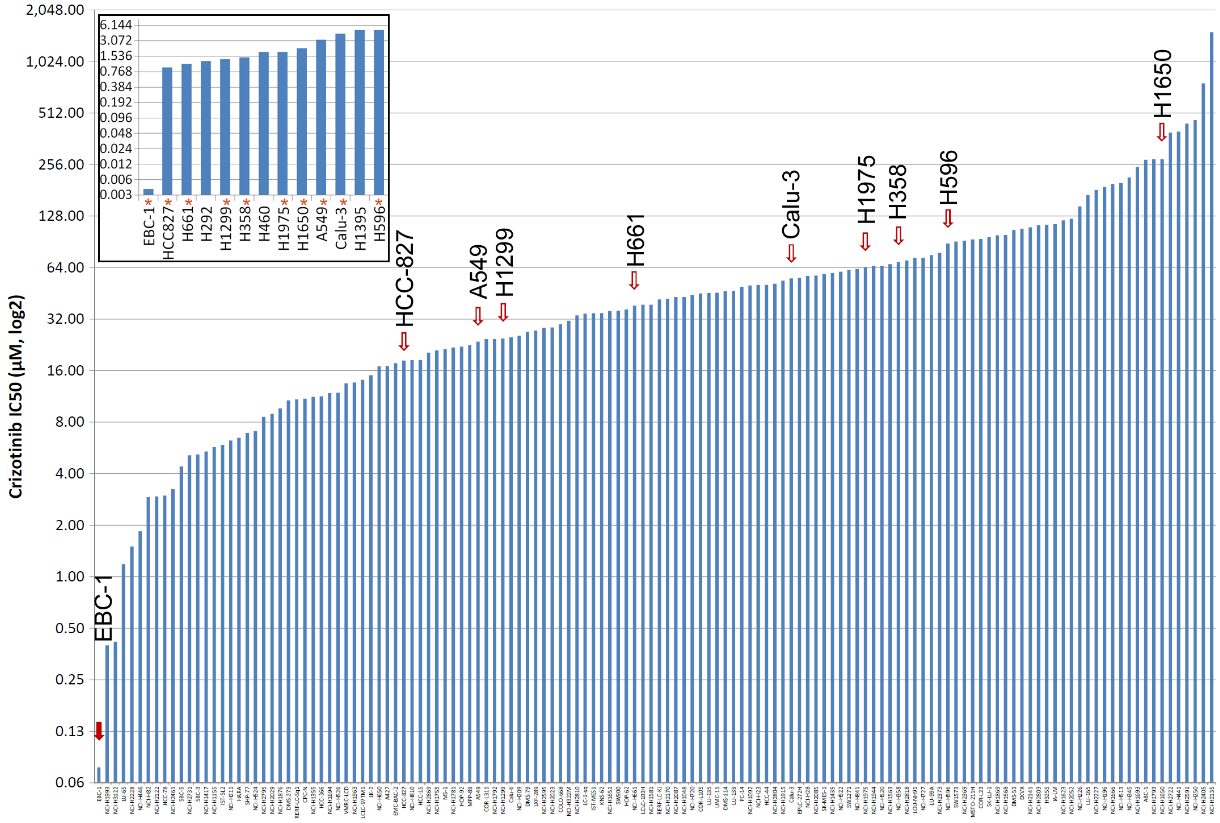

Figure S1. Crizotinib IC50 values for various cancer cell lines in Pharmacogenomics datasets of the Cancer Cell Line Encyclopedia (CCLE) project [27]. Crizotinib IC50 values for 13 NSCLC cell lines detected in the present study were also displayed in the inserted chart. Red-star and red-arrow, 10 cell lines tested in both the CCLE and present studies

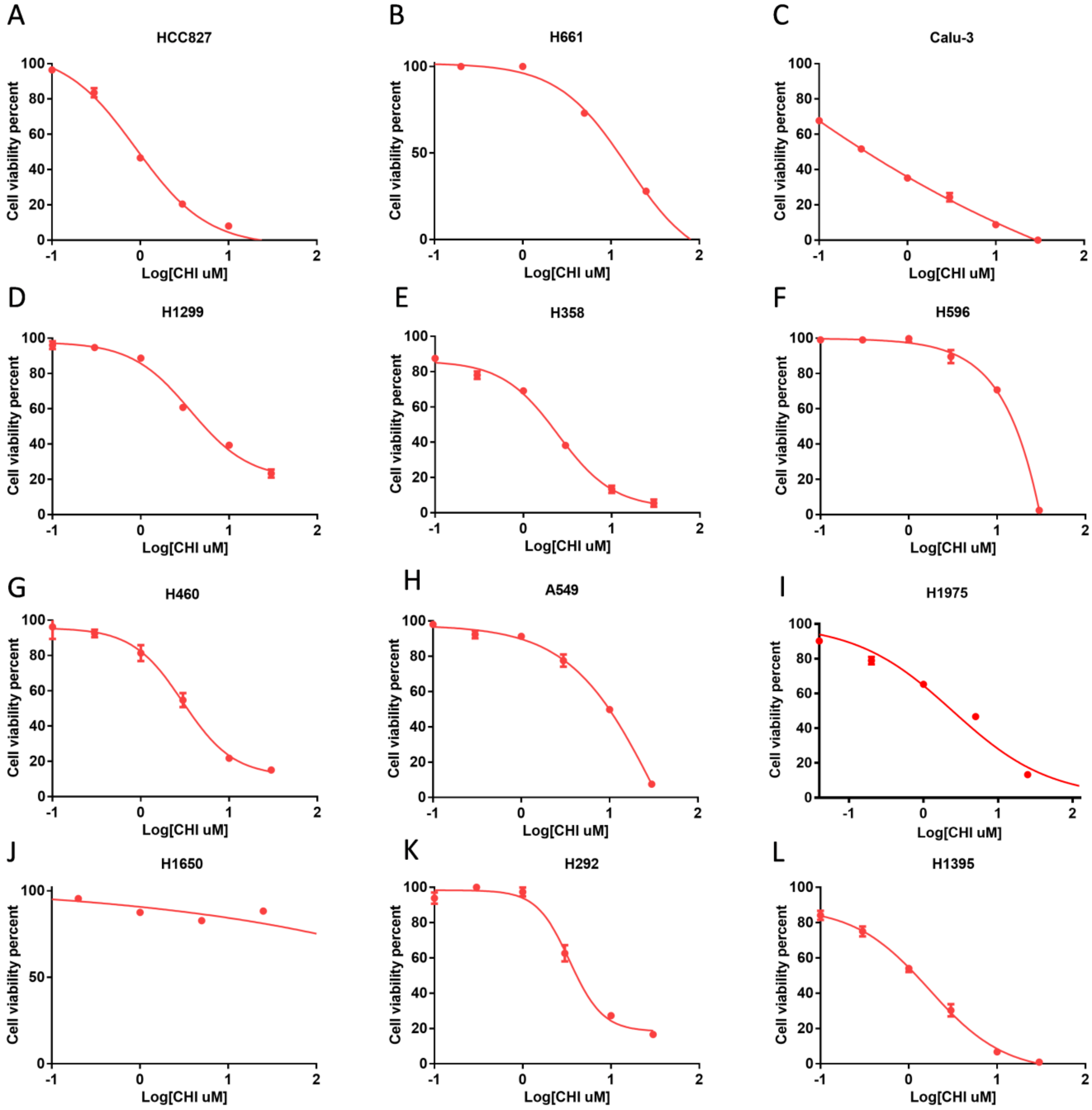

Figure S2. Cell viability curves of 12 NSCLC cell lines treated with chidamide (CHI) at various concentrations for 72 hr. (**A**) HCC827; (**B**) H661; (**C**) Calu-3; (**D**) H1299; (**E**) H358; (**F**) H596; (**G**) H460; (**H**) A549; (**I**) H1975; (**J**) H1650; (**K**) H292; (**L**) H1395.

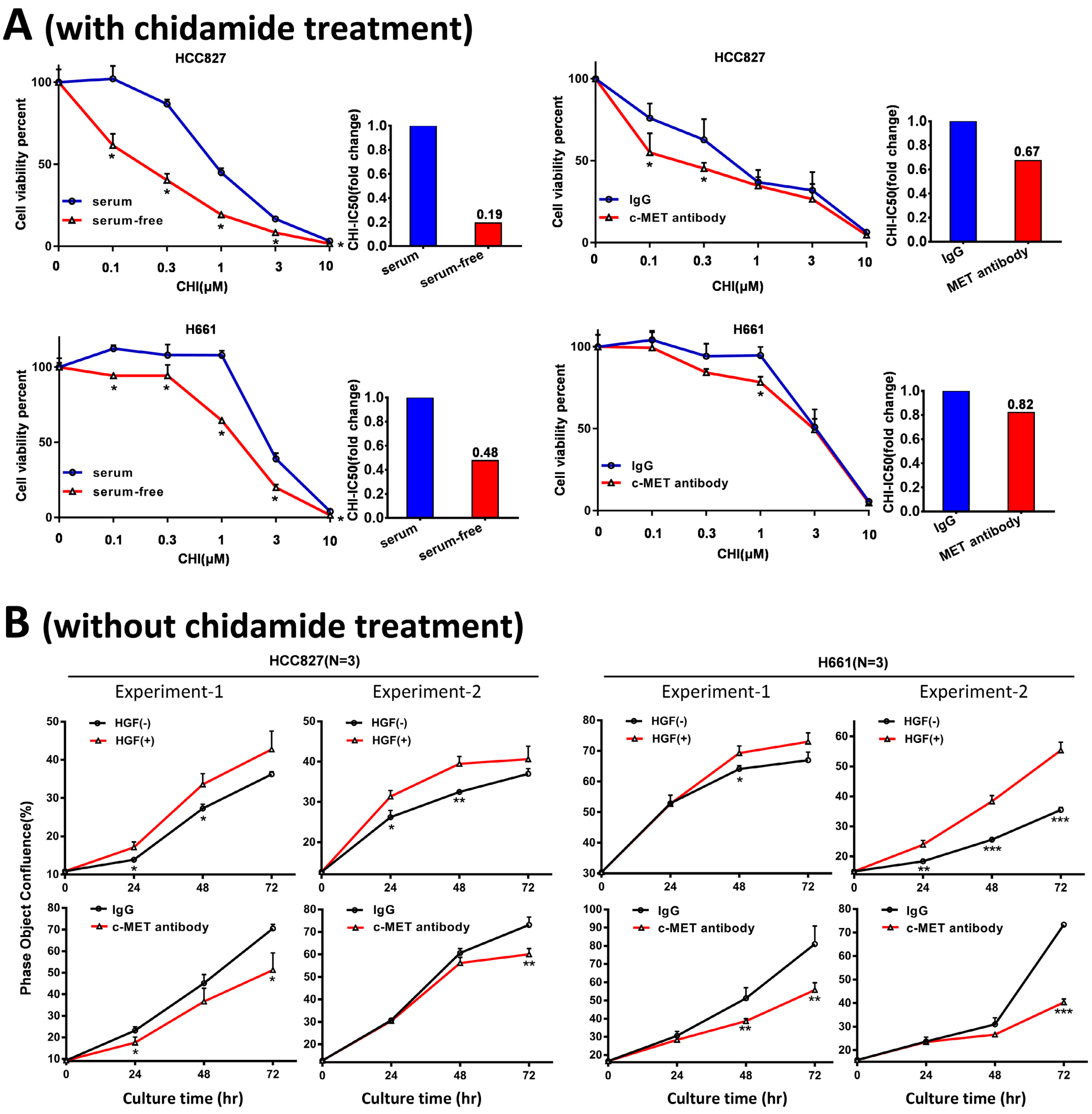

Figure S3. Effect of loss of function of *c-MET* by antibody (2.5 μg/mL) against c-MET or HGF-deprivation (serum-free) on the proliferation/viability of HCC827 and H661 cells treated with and without various concentrations of chidamide (CHI). The experimental conditions were the same as described in Figure 4 legend. (**A**) With chidamide treatment; The fold change of chidamide IC50 for cells treated with c-MET antibody or cultured in serum-free medium (HGF-deprivation) relative to cells treated with IgG control or cultured in serum-containing medium was labeled in right chart, respectively. (**B**) Without chidamide treatment. The results of two repeat experiments were illustrated. HGF(-),HGF-deprivation (serum-free); HGF(+), serum-free medium with HGF supplement; */**/***, P <0.05/0.01/0.001

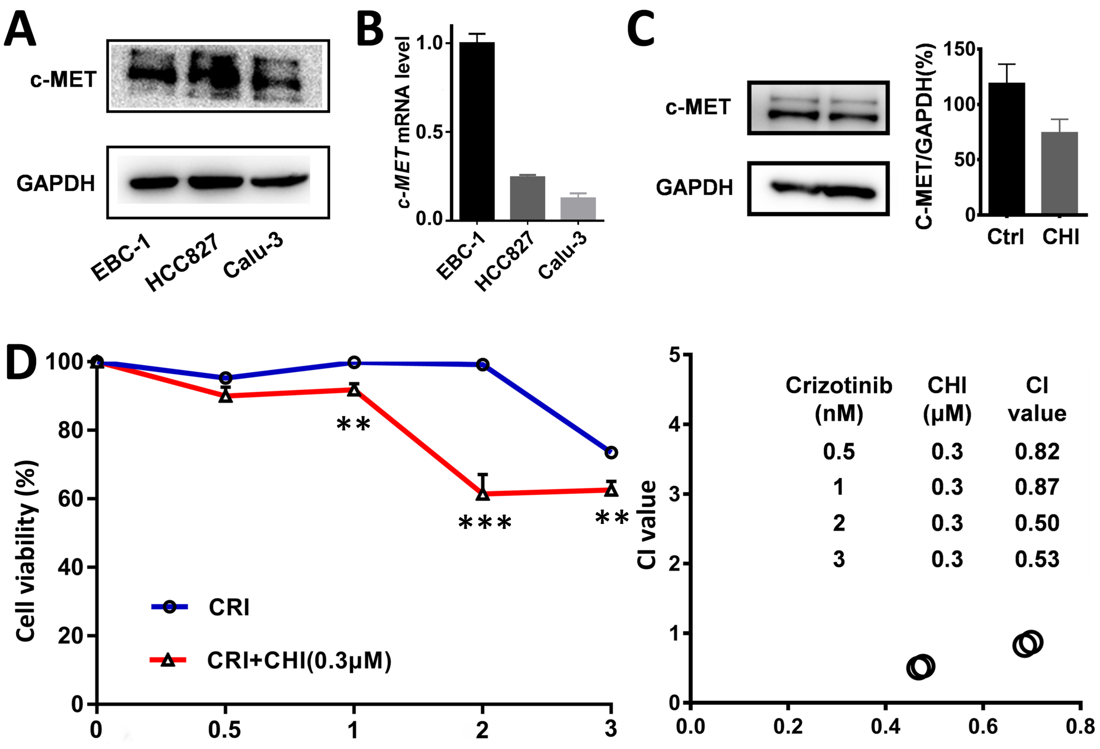

Figure S4. Synergistic effect of chidamide-crizotinib in NSCLC cell line EBC-1 with *c-MET* gene amplification. (**A** and **B**) The levels of baseline c-MET protein and mRNA in EBC-1 and other cell lines in Western blot and quantitative RT-PCR analyses, respectively. (**C**) The level of c-MET protein in EBC-1 cells with treatment of chidamide (CHI, at 1/4 IC50 [0.3μM]) for 72 hr in Western blotting. (**D**) Cell viability was measured by using the IncuCyte platform (left chart). The crizotinib-IC50 values for EBC-1 cells were calculated in the absence or presence of chidamide. The synergetic effect of chidamide-crizotinib cotreatment on cell proliferation inhibition was calculated using the CI equation and presented as Fa (fraction affected by the dose) in the Fa–CI plots (right chart); */**/***, P <0.05/0.01/0.001

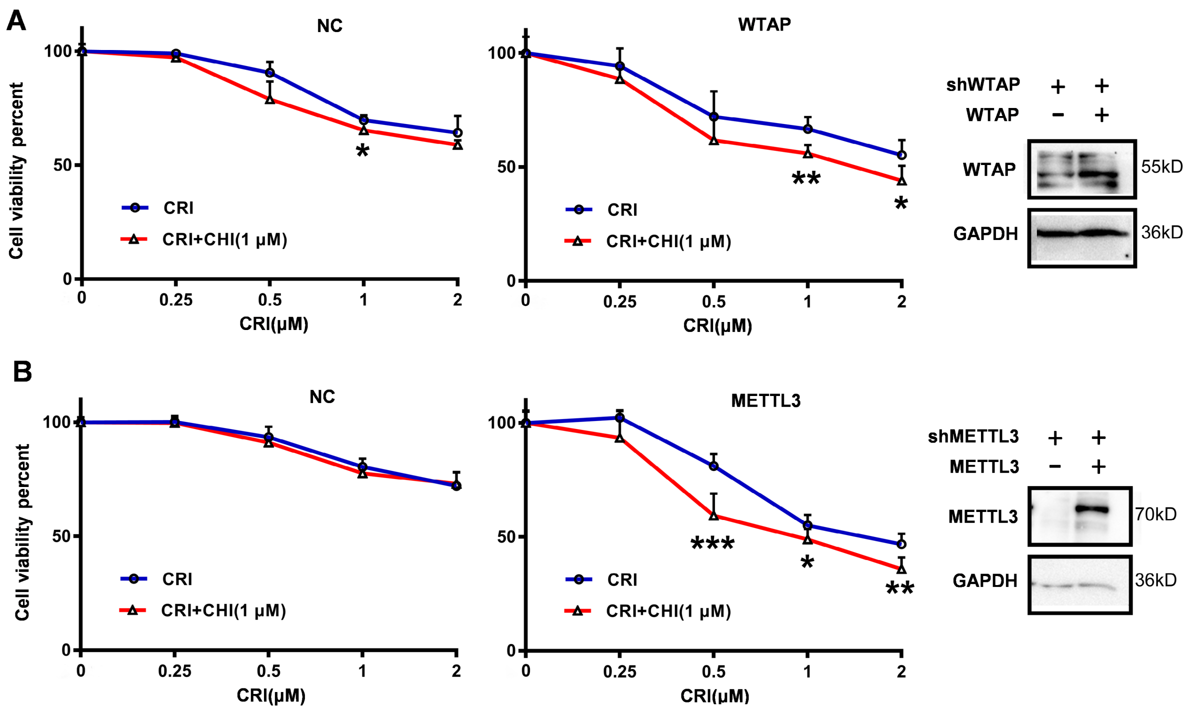

Figure S5. Effect of *WTAP* or *METTL3* overexpression on the synergetic effect of chidamide-crizotinib in H661 cells with shWTAP or shMETTL3 transfection in the rescue assays. (**A**) *WTAP* overexpression in cells with stable *WTAP* knockdown by shRNA transfection. (**B**) *METTL3* overexpression in cells with stable *METTL3* knockdown by shRNA transfection. The results of Western blotting were inserted in the right side to monitor changes of the amounts of WTAP or METTL3 proteins in these cells. */**/***, *P* <0.05/0.01/0.001

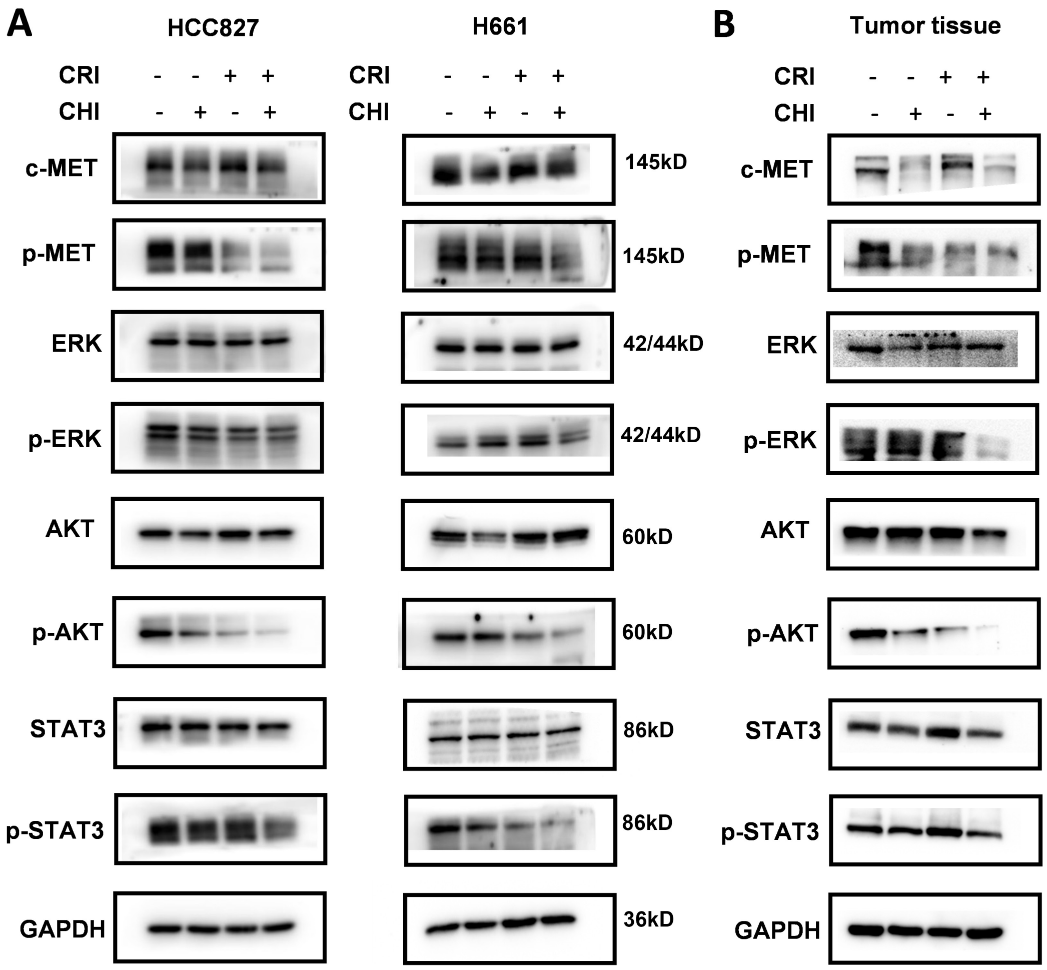

Figure S6. Effects of treatment with chidamide (CHI), crizotinib (CRI), or their combination on the phosphorylation levels of the receptor tyrosine kinase (RTK) signaling molecules c-MET, ERK, AKT and STAT3 proteins in NSCLC cells via Western blotting. (**A**) The NSCLC cell lines HCC827 and H661 treated with chidamide (0.25 μM for HCC827 and 1 μM for H661), crizotinib (1 μM), or their combination for 4 hr. (**B**) HCC827-derived tumors in mice treated with chidamide (5 mg/kg/d), crizotinib (25 mg/kg/d), or their combination for 21 days.

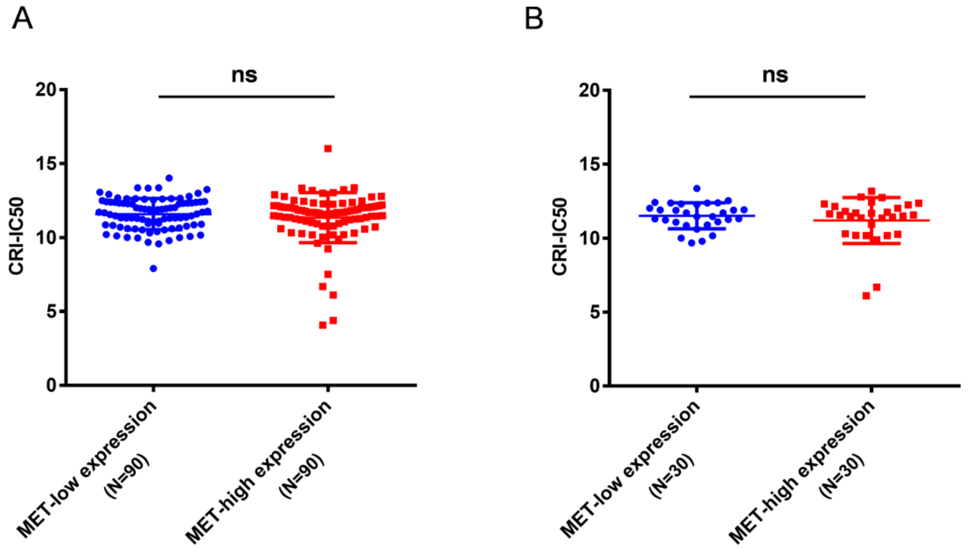

Figure S7. The IC50 values of crizotinib in human cancer cell lines with different expression statuses of *c-MET* mRNA in the Cancer Cell Line Encyclopedia (CCLE) project [26]. The cell lines were equally stratified into the high *c-MET* expression and low *c-MET* expression groups. (**A**) All 180 human cancer cell lines. (**B**) 60 lung cancer cell lines. ns, not significant.

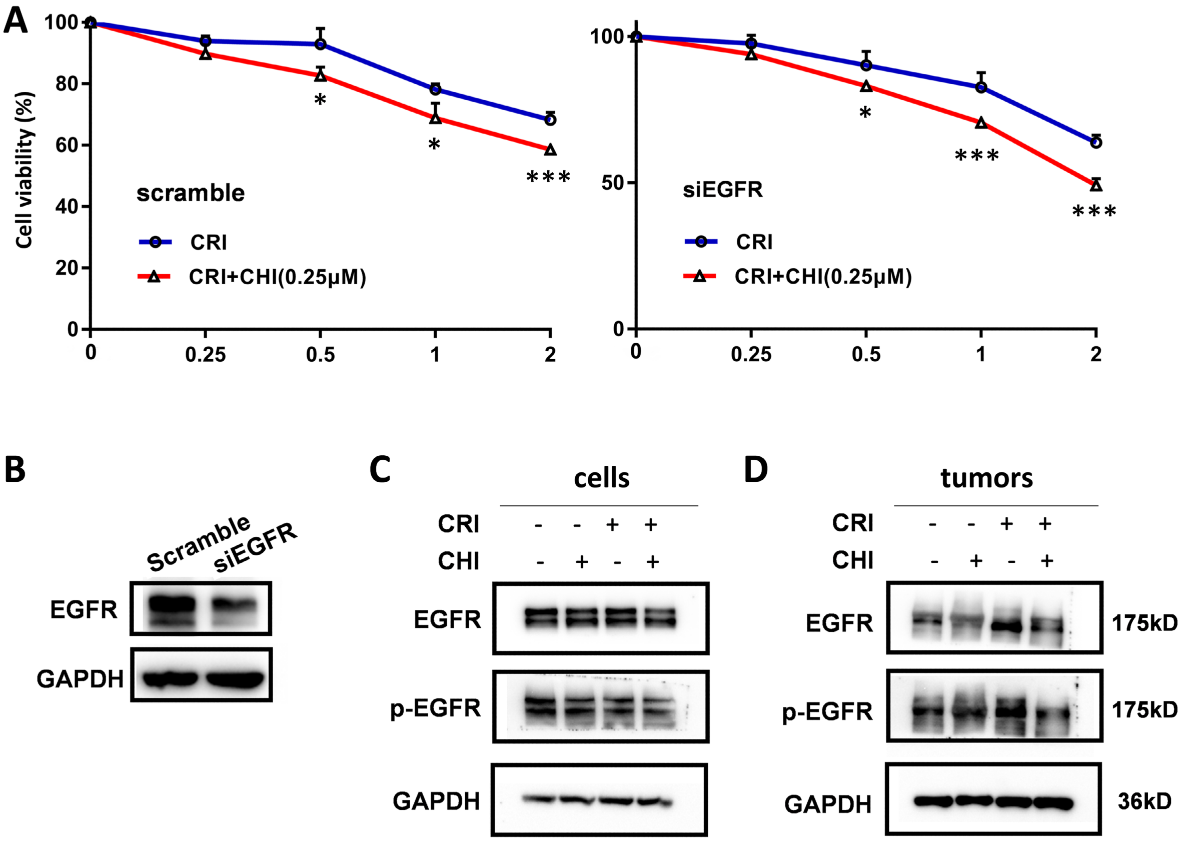

Figure S8. Effect of *EGFR* downregulation by siRNA on the viability and EGFR phosphorylation of HCC827 cells with treatment of chidamide, crizotinib, and their combination. (**A**) Synergistic effect of chidamide-crizotinib in HCC827 cells with and without siRNA knockdown of *EGFR* expression (siEGFR). The crizotinib-IC50 values for EBC-1 cells were calculated in the absence or presence of chidamide. (**B**) The level of global EGFR protein in HCC827 cells 48 hr posttransfection in Western blotting. (**C**) The levels of global EGFR and phosphorylated EGFR (pEGFR) proteins in HCC827 cells treated with chidamide (0.25 μM), crizotinib (1 μM), or their combination for 4 hr in Western blotting; (**D**) The level of global EGFR protein in HCC827-derived tumors in mice treated with chidamide (5 mg/kg/d), crizotinib (25 mg/kg/d), or their combination for 21 days. */**/***, $P < 0.05/0.01/0.001$

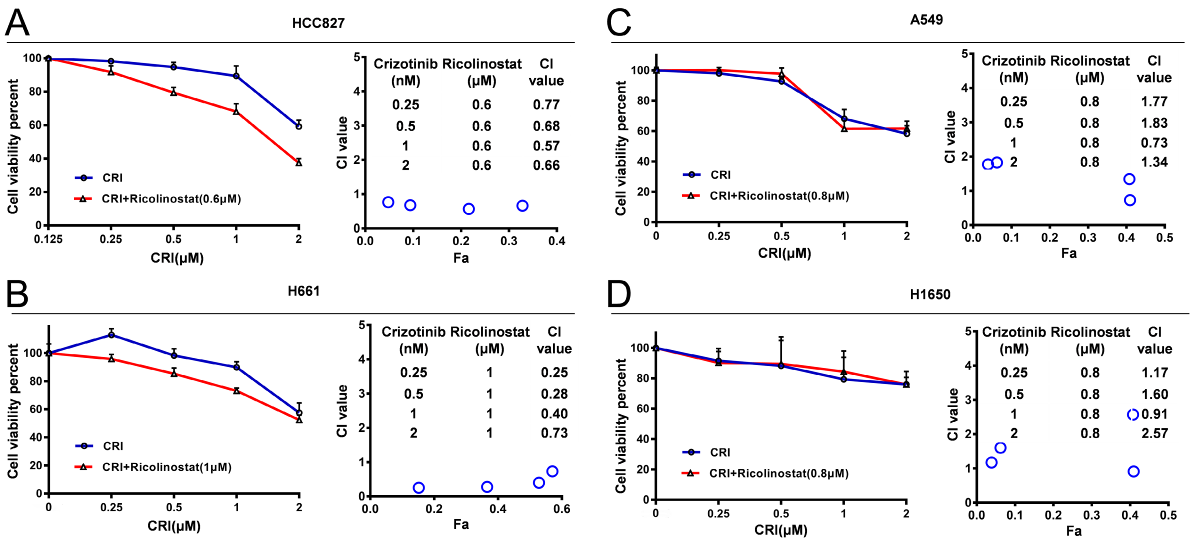

Figure S9. Effect of ricolinostat on the ALK mutation-free NSCLC cells to crizotinib. NSCLC cells were treated with various concentrations of crizotinib (CRI) alone or combined with ricolinostat at ≤1/4 IC50 for 72 hr. Cell viability was measured by using the IncuCyte platform (left charts). The synergetic effect of ricolinostat-crizotinib cotreatment on cell proliferation inhibition was calculated using the CI equation and presented as Fa (fraction affected by the dose) in the Fa–CI plots (right chart). (**A**) HCC827; (**B**) H661; (**C**) A549; (**D**) H1650.

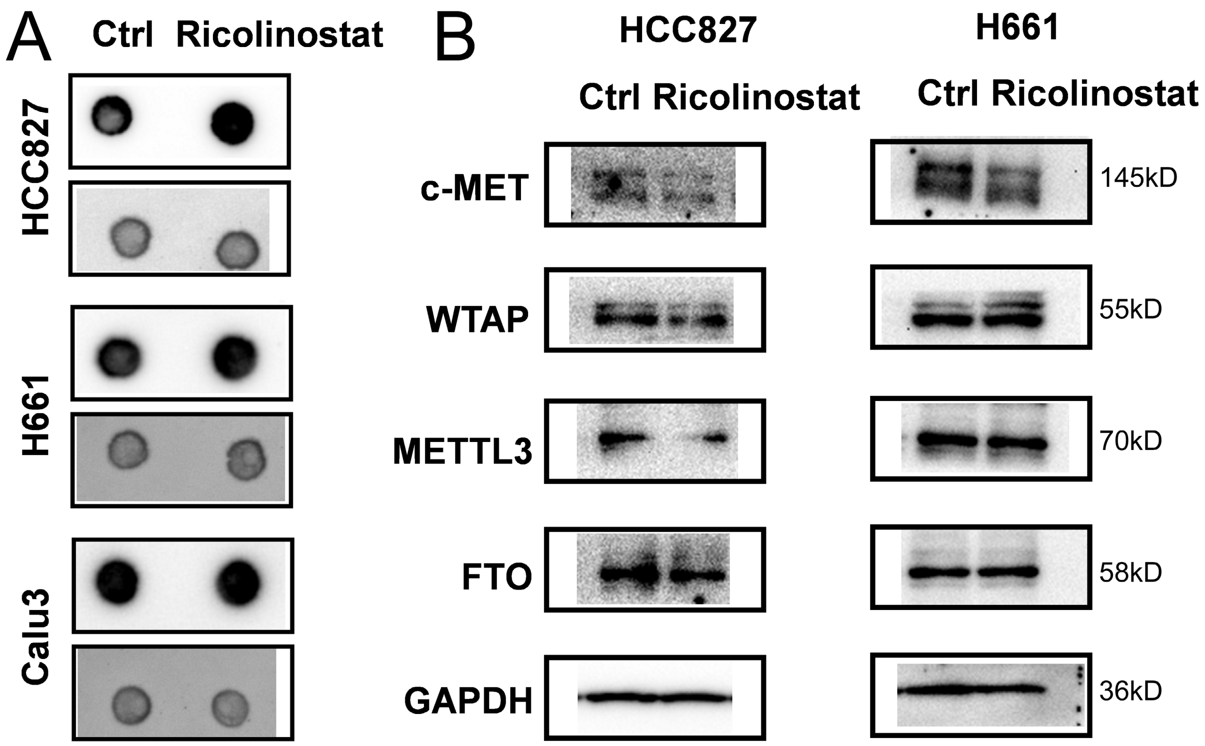

Figure S10. Effect of ricolinostat on RNA m6A methylation of NSCLC cells. (**A**) The amounts of total m6A RNA of ricolinostat-crizotinib-sensitive cell lines (HCC827, H661, and Calu3) treated with ricolinostat (0.6 μM for HCC827, 1 μM for H661 and 0.5 μM for Calu-3; 4 hr) in dot blotting analyses. (**B**) The levels of c-MET, WTAP, METTL3, and FTO proteins in ricolinostat-treated HCC827 and H661 cells in Western blotting
